# Evaluation of Resistance in Bt Maize Event DBN3601T Expressing Cry1Ab and Vip3Aa Proteins Against *Athetis lepigone* (Möschler) in North China

**DOI:** 10.3390/plants15111669

**Published:** 2026-05-29

**Authors:** Zhenghao Zhang, Zhizhang Gong, Guodong Kang, Xianming Yang, Youming Hou, Kongming Wu

**Affiliations:** 1 State Key Laboratory of Agricultural and Forestry Biosecurity, Fujian Agriculture and Forestry University, Fuzhou 350002, China; zhenghaozzh@163.com (Z.Z.); ymhou@fafu.edu.cn (Y.H.); 2 State Key Laboratory for Biology of Plant Diseases and Insect Pests, Institute of Plant Protection, Chinese Academy of Agricultural Sciences, Beijing 100193, China; 18973281861@163.com (Z.G.); kanggd95@163.com (G.K.); yangxianming@caas.cn (X.Y.); 3 National Center of Technology Innovation for Comprehensive Utilization of Saline-Alkali Land, Dongying 257300, China

**Keywords:** Bt maize, Cry1Ab, Vip3Aa, *Athetis lepigone*, tissue-specific expression, larval susceptibility, host plant resistance

## Abstract

*Athetis lepigone* (Möschler) is an important pest of maize in North China, whose larvae feed mainly on maize leaves, stems, and roots during the seedling stage, with conventional maize lacking effective resistance to it. In recent years, transgenic Bt maize expressing Cry1Ab and Vip3Aa proteins has been commercialized in China; however, its resistance against *A. lepigone* has not yet been systematically evaluated. In this study, three Bt maize events, DBN3601T expressing Cry1Ab and Vip3Aa, DBN9936 expressing Cry1Ab, and DBN9501 expressing Vip3Aa, were used to comprehensively assess resistance against the pest based on Bt protein expression levels in different maize tissues, larval susceptibility across instars, and larval feeding behavior under controlled laboratory conditions. The results showed that Bt protein expression varied significantly among maize tissues commonly fed upon by the insect, following the general pattern: seedling leaf > stem > root. Bioassays using artificial diets incorporated with freeze-dried maize leaf powder indicated that larvae were significantly more susceptible to Cry1Ab than to Vip3Aa, with LC_50_ values of 1.05 and 2.65 μg·g^−1^, respectively. Maize co-expressing both proteins exhibited high insecticidal activity. First-instar larvae displayed feeding avoidance of Bt maize tissues, and early instars were more sensitive than later instars; however, stems and roots showed stronger toxicity to older larvae. In simulated field infestation assays, the control efficacies of DBN3601T, DBN9936, and DBN9501 reached 94.35%, 88.79%, and 10.56%, respectively, at five days post-infestation. Overall, DBN3601T maize exhibited a strong resistance performance against *A. lepigone*, indicating strong potential for pest management applications.

## 1. Introduction

Maize is one of the three major cereal crops worldwide and ranks first in global production, playing a fundamental role in food security, feed supply, and industrial raw material production [1]. However, insect pests are among the most important biotic constraints limiting stable and high maize yields. Globally, yield losses caused by pests and diseases are estimated to range from 19.5% to 41.1% annually, posing a serious threat to food supply [2]. *Athetis lepigone* (Möschler) (Lepidoptera: Noctuidae) has emerged as an increasingly important maize pest in North China, the Huang–Huai–Hai region, and Northeast China in recent years. It was first reported to damage summer maize seedlings in Hebei Province in 2005 and rapidly expanded to form a stable infestation area within a decade [3,4]. This species is widely distributed across Eurasia [5,6,7]. Early instar larvae mainly feed on leaves and young tissues, whereas late instars attack stems and roots, often remaining concealed at the soil surface or near the plant base, resulting in stand loss and suppressed plant growth [8,9,10]. This basal feeding habit distinguishes *A. lepigone* from typical foliar-feeding pests and suggests a distinct interaction between the insect and its host plant. Understanding the relationship between host resistance expression and pest feeding characteristics is therefore essential for elucidating damage mechanisms and improving control strategies.

Host plant resistance is a key component of sustainable pest management. Conventional maize varieties generally lack stable and effective resistance against *A. lepigone*, which may be insufficient under high pest pressure [11,12,13]. With advances in molecular breeding, transgenic crops expressing insecticidal proteins derived from *Bacillus thuringiensis* (Bt) have substantially enhanced resistance to target pests [14]. Bt crops can suppress pest populations, reduce chemical pesticide use, increase farmers’ economic returns, and help conserve natural enemies in agroecosystems [15,16]. In Bt maize, resistance to lepidopteran and coleopteran pests is mainly mediated by Cry and Vip proteins [17,18]. These proteins differ in their modes of action and generally show no cross-resistance [19,20], leading to marked variation in toxicity among target pests. Such variation is largely associated with differences in midgut receptor composition, toxin-binding specificity, and toxin activation processes among insect species. For example, Cry proteins are highly effective against *Ostrinia furnacalis* (Guenée) [21], whereas Vip proteins exhibit greater activity against *Spodoptera frugiperda* (J. E. Smith) [22]. In addition to protein type, Bt protein expression varies significantly among maize tissues, typically being higher in leaves [23]. This spatial heterogeneity results in uneven resistance distribution within the plant and may influence the actual exposure of herbivorous insects to Bt toxins. This is particularly relevant for *A. lepigone*, because early instars mainly feed on leaves and young tissues, whereas later instars increasingly attack stems and roots. Therefore, the effectiveness of Bt maize against *A. lepigone* may depend on the spatial match between tissue-specific Bt protein expression and larval feeding sites.

In recent years, the global cultivation area of transgenic crops has continued to expand, and Bt maize has been widely adopted. By 2024, the global planting area of Bt maize had reached approximately 68.4 million hectares [24]. In China, multiple Bt maize events, approximately 25, have obtained biosafety certificates and are being commercially cultivated across different agroecological regions [25]. Previous studies have demonstrated that Bt maize provides effective control against various lepidopteran pests, including *S. frugiperda*, *Helicoverpa armigera*, *O. furnacalis*, and *Conogethes punctiferalis* [26]. In particular, pyramided Bt maize expressing both Cry and Vip proteins shows clear advantages in broadening the insecticidal spectrum and delaying resistance evolution, because these proteins differ in their modes of action and generally show limited cross-resistance [27]. However, studies on *A. lepigone* remain limited. In particular, the relationship between Bt protein expression patterns in maize and resistance to this pest remains unclear. Moreover, how differences in protein type and tissue-specific expression jointly influence resistance has not been systematically investigated.

In this study, three Bt maize events, DBN3601T (Cry1Ab + Vip3Aa), DBN9936 (Cry1Ab), and DBN9501 (Vip3Aa) were used to systematically compare Bt protein expression patterns and insecticidal efficacy from the perspective of host plant resistance. By integrating Bt protein quantification, bioassays, tissue-specific resistance evaluation, feeding behavior analysis, and simulated field cage experiments, this study aimed to comprehensively assess the resistance of different Bt maize events to *A. lepigone* and to clarify the combined effects of protein type and tissue-specific expression on resistance. These findings may help optimize the use of Bt maize in sustainable pest management.

## 2. Results

### 2.1. Comparison of Bt Protein Expression in Maize Tissues Targeted by Athetis lepigone

Bt proteins were detected in the leaves, stems, and roots of all three transgenic maize events, DBN3601T, DBN9936, and DBN9501. For Cry1Ab, two-way ANOVA revealed no significant effect of maize event, whereas tissue type had a significant effect on protein expression (*F*_2,12_ = 23.73, *p* < 0.0001). In contrast, Vip3Aa expression differed significantly among maize events (*F*_2,12_ = 6.51, *p* = 0.0254) and among tissues (*F*_2,12_ = 111.10, *p* < 0.0001). For total Bt protein, both maize event (*F*_2,18_ = 393.10, *p* < 0.0001) and tissue type (*F*_2,18_ = 157.00, *p* < 0.0001) had significant main effects, and a significant interaction between these two factors was also detected (*F*_4,18_ = 9.93, *p* = 0.0002). Simple effects analysis further showed that total Bt protein expression consistently followed the pattern leaves > stems > roots across all maize events (Table 1). Among the three events, total Bt protein levels were highest in DBN3601T, followed by DBN9936 and DBN9501.

### 2.2. Differential Susceptibility of Athetis lepigone Larvae to Bt Proteins Expressed in Three Maize Events

The results showed that *A. lepigone* larvae were more susceptible to Cry1Ab than to Vip3Aa (Table 2). The LC_50_ value for Cry1Ab expressed in DBN9936 was 1.05 μg∙g^−1^, whereas that for Vip3Aa expressed in DBN9501 was 2.65 μg∙g^−1^. The pyramided Bt maize event DBN3601T, expressing Cry1Ab + Vip3Aa, had an LC_50_ value of 2.33 μg∙g^−1^.

### 2.3. Insecticidal Effects of Different Bt Maize Tissues on Athetis lepigone Larvae

**Figure 1:**
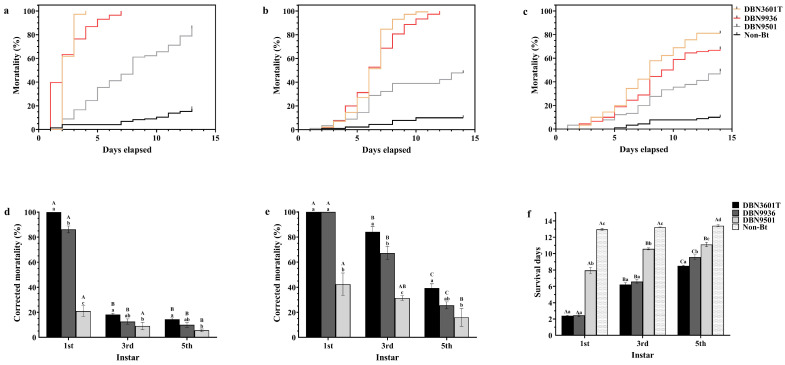
Mortality dynamics of *Athetis lepigone* larvae fed leaves of different Bt maize events. (**a**–**c**) Cumulative mortality of first-, third-, and fifth-instar larvae, respectively. (**d**,**e**) Corrected mortality at 4 and 7 days after treatment. (**f**) Larval survival time. Survival curves in (**a**–**c**) were generated using the Kaplan–Meier method, and differences among treatments were compared using the log-rank (Mantel–Cox) test (*p* < 0.05). Data in (**d**–**f**) were analyzed using two-way ANOVA followed by Tukey’s multiple-comparison test. Different lowercase letters indicate significant differences among maize treatments within the same larval instar, whereas different uppercase letters indicate significant differences among larval instars within the same maize treatment (*p* < 0.05).

Larval mortality increased with feeding duration when *A. lepigone* larvae were fed on maize leaves. Kaplan–Meier survival analysis followed by log-rank tests revealed significant differences in survival curves among maize treatments for first instars (*χ*^2^ = 501.0, *df* = 3, *p* < 0.0001; Figure 1a), third instars (*χ*^2^ = 286.8, *df* = 3, *p* < 0.0001; Figure 1b), and fifth instars (*χ*^2^ = 102.2, *df* = 3, *p* < 0.0001; Figure 1c). Larvae fed on DBN9936 and DBN3601T showed the fastest mortality, with no significant difference between these two events, whereas mortality was significantly slower in DBN9501. The non-Bt control exhibited the lowest mortality. At 4 and 7 days after feeding, corrected mortality differed significantly among Bt maize events (day 4: *F*_2,18_ = 634.0, *p* < 0.0001; day 7: *F*_2,18_ = 101.5, *p* < 0.0001) and among larval instars (day 4: *F*_2,18_ = 163.4, *p* < 0.0001; day 7: *F*_2,18_ = 101.5, *p* < 0.0001). Corrected mortality followed the pattern DBN3601T > DBN9936 > DBN9501 and decreased with increasing larval instar (Figure 1d,e). Significant differences were also observed in larval survival time among maize treatments (*F*_3,24_ = 919.6, *p* < 0.0001) and among larval instars (*F*_2,24_ = 453.7, *p* < 0.0001). Larvae fed on non-Bt maize had the longest survival time, whereas those fed on DBN3601T and DBN9936 showed significantly shorter survival (5.71 and 6.20 days, respectively) compared with DBN9501 (9.88 days). In addition, survival time increased with larval instar (Figure 1f).

**Figure 2:**
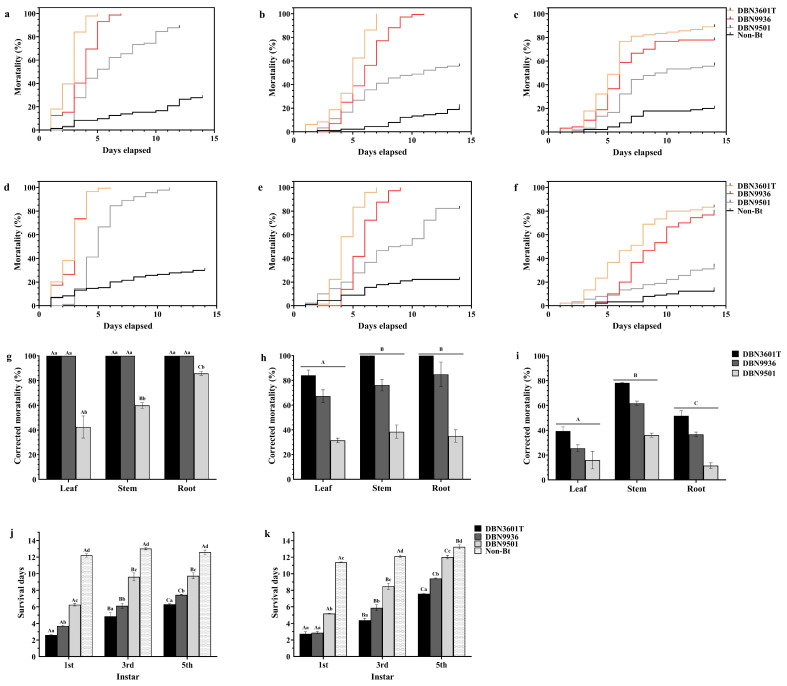
Insecticidal effects of stem and root tissues of different Bt maize events on *Athetis lepigone* larvae. (**a**–**c**) Mortality dynamics of first-, third-, and fifth-instar larvae fed stems. (**d**–**f**) Mortality dynamics of first-, third-, and fifth-instar larvae fed roots. (**g**–**i**) Corrected mortality at 7 days after feeding on different maize tissues. (**j**,**k**) Larval survival time after feeding on stems and roots, respectively. Mortality curves in (**a**–**f**) were generated from Kaplan–Meier estimates, and differences among treatments were compared using the log-rank (Mantel–Cox) test (*p* < 0.05). Data in (**g**–**k**) were analyzed using two-way ANOVA followed by Tukey’s multiple-comparison test. No significant interaction between maize event and tissue type was detected in (**h**) or (**i**) (*p* > 0.05). Different uppercase letters indicate significant differences among tissues within the same maize event, whereas different lowercase letters indicate significant differences among maize events within the same tissue (*p* < 0.05).

During the 14-day feeding period, Kaplan–Meier estimates followed by log-rank tests revealed significant differences in survival curves among maize treatments when larvae were fed stems (first instar: *χ*^2^ = 437.4, *df* = 3, *p* < 0.0001, Figure 2a; third instar: *χ*^2^ = 296.4, *df* = 3, *p* < 0.0001, Figure 2b; fifth instar: *χ*^2^ = 118.6, *df* = 3, *p* < 0.0001, Figure 2c) and roots (first instar: *χ*^2^ = 390.9, *df* = 3, *p* < 0.0001, Figure 2d; third instar: *χ*^2^ = 227.5, *df* = 3, *p* < 0.0001, Figure 2e; fifth instar: *χ*^2^ = 146.5, *df* = 3, *p* < 0.0001, Figure 2f). Within 11 days, mortality of first and third-instar larvae fed stems and roots of DBN3601T and DBN9936 reached 100% (Figure 2a,b,d,e). For fifth-instar larvae, mortality increased in the order DBN3601T > DBN9936 > DBN9501 > non-Bt maize (Figure 2c,f). Larval survival time differed significantly among maize treatments for both stems (*F*_3,24_ = 570.4, *p* < 0.0001) and roots (*F*_3,24_ = 675.4, *p* < 0.0001) (Figure 2j), as well as among larval instars (stems: *F*_2,24_ = 132.3, *p* < 0.0001; roots: *F*_2,24_ = 542.1, *p* < 0.0001) (Figure 2k). Larvae fed stems and roots of DBN3601T and DBN9936 had significantly shorter survival times than those fed DBN9501, and survival time increased with larval instar.

At 7 days after feeding, corrected mortality differed significantly among maize tissues (Figure 2g–i). Two-way ANOVA revealed a significant interaction between tissue type and maize event for first-instar larvae (*F*_4,18_ = 16.33, *p* < 0.0001), whereas no significant interaction was detected for third- or fifth-instar larvae (*p* > 0.05). For first instars, corrected mortality differed significantly among all treatment combinations (Figure 2g), with all Bt maize events showing high toxicity and DBN3601T and DBN9936 causing significantly higher mortality than DBN9501. For third- and fifth-instar larvae, both tissue type and maize event had significant effects on corrected mortality (*p* < 0.05). Mortality was significantly higher in those fed stems and roots than in those fed leaves and followed the order DBN3601T > DBN9936 > DBN9501 (Figure 2h,i). Overall, larval susceptibility to Bt maize decreased with increasing instar. However, tissue-specific differences in toxicity remained consistent in later instars, with stems and roots showing stronger insecticidal effects than leaves. This pattern may be related to the feeding sites and tissue utilization of older larvae, which preferentially attack basal stem and root tissues and may consequently receive greater effective exposure to Bt proteins in these tissues.

### 2.4. Feeding Preference of Athetis lepigone Larvae for Bt and Non-Bt Maize

**Figure 3:**
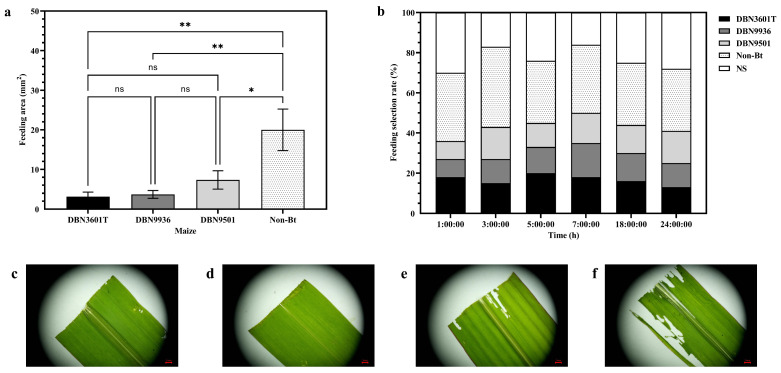
Feeding preference of *Athetis lepigone* larvae among different maize treatments. (**a**) Consumed leaf area after 24 h. (**b**) Feeding selection rate (%) over time. (**c**–**f**) Representative images of leaf damage after larval feeding on DBN3601T, DBN9936, DBN9501, and non-Bt maize leaves, respectively. “NS” indicates no selection. Data are presented as means ± SE. Consumed leaf area in (**a**) was analyzed using one-way ANOVA followed by Tukey’s multiple-comparison test. Feeding selection rate in (**b**) was analyzed using two-way ANOVA to assess the effects of maize treatment and time, followed by Tukey’s multiple-comparison test. Asterisks indicate significant differences among treatments (* *p* < 0.05; ** *p* < 0.01), and “ns” indicates no significant difference.

Feeding choice assays revealed significant differences in consumed leaf area among maize treatments (*F*_3,36_ = 6.973, *p* = 0.0008). The mean consumed leaf areas on DBN3601T, DBN9936, DBN9501, and non-Bt maize were 3.15, 3.71, 7.33, and 19.99 mm^2^, respectively. Larvae consumed significantly more leaf tissue from non-Bt maize than from Bt maize, whereas no significant differences were detected among the three Bt maize events (Figure 3a). Similarly, feeding selection rates differed significantly among maize treatments (*F*_4,270_ = 27.64, *p* < 0.0001). Within 24 h, the mean selection rates for DBN3601T, DBN9936, DBN9501, and non-Bt maize were 16.67%, 12.83%, 13.67%, and 33.50%, respectively. No significant differences were observed among the Bt maize events, whereas larvae showed a significantly higher preference for non-Bt maize (Figure 3b).

### 2.5. Population Dynamics and Control Efficacy of Bt Maize Against Athetis lepigone

**Figure 4:**
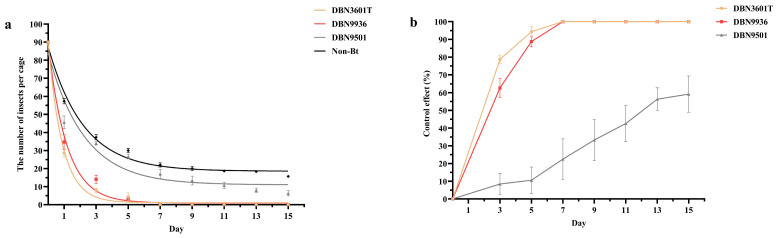
Control efficacy of Bt maize against *Athetis lepigone* under controlled cage conditions. (**a**) Population dynamics of larvae in cage experiments under different maize treatments. (**b**) Control efficacy of different maize treatments. In (**a**), larval population dynamics were fitted using a one-phase exponential decay model, showing a good fit across treatments (*R*^2^ = 0.936–0.992). Differences among fitted curves were compared using the extra sum-of-squares F-test (*p* < 0.05). In (**b**), control efficacy was analyzed using two-way ANOVA followed by Tukey’s multiple comparison test (*p* < 0.05). Data are presented as mean ± SE.

Nonlinear regression analysis showed that population dynamics differed significantly among treatments (extra sum-of-squares F-test, *p* < 0.0001). In all treatments, larval populations followed an exponential decay pattern, although the rate of decline varied markedly. The decay rate constant (*k*) was higher in DBN3601T (*k* = 1.119) and DBN9936 (*k* = 0.8595) than in DBN9501 (*k* = 0.4431) and the non-Bt control (*k* = 0.4537), indicating a significantly faster population decline under DBN3601T and DBN9936 treatments. Half-life analysis further confirmed significant differences in population decline among maize treatments. The half-lives of DBN3601T and DBN9936 were 0.62 d and 0.81 d, respectively, which were significantly shorter than those of DBN9501 (1.56 d) and the non-Bt control (1.53 d). Notably, no significant difference was observed between DBN9501 and the non-Bt control in terms of population reduction (Figure 4a), suggesting that Vip3Aa alone may provide limited control efficacy against *A. lepigone* under the simulated cage conditions. Control efficacy differed significantly among Bt maize events (*F*_2,48_ = 278.1, *p* < 0.0001), with no significant difference between DBN3601T and DBN9936. At 5 days after treatment, the control efficacies of DBN3601T, DBN9936, and DBN9501 were 94.35%, 88.79%, and 10.56%, respectively. By day 7, complete mortality of *A. lepigone* larvae was observed in DBN3601T and DBN9936 treatments, resulting in 100% control efficacy, whereas DBN9501 achieved only 22.50% (Figure 4b).

## 3. Discussion

This study systematically evaluated the resistance performance of Bt maize expressing Cry1Ab and Vip3Aa by integrating tissue-specific expression patterns with biological responses of *A*. *lepigone*. The results demonstrate that Bt protein expression varied significantly among maize tissues, with consistently higher levels in leaves than in stems and roots. In addition, differences in expression levels were observed among maize events, indicating that spatial expression patterns of Bt proteins constitute a fundamental determinant of Bt maize resistance performance. Beyond expression patterns, the *A. lepigone* population tested in this study exhibited differential susceptibility to Bt toxins, showing higher sensitivity to Cry1Ab than to Vip3Aa. Consequently, maize events expressing Cry1Ab (DBN3601T and DBN9936) displayed stronger insecticidal performance. Moreover, larvae showed partial feeding avoidance of Bt maize, suggesting that resistance is not solely determined by toxicity but may also involve behavioral responses that alter feeding patterns. Taken together, these findings indicate that the resistance of Bt maize is jointly determined by protein expression patterns, toxin type, and insect behavioral responses.

Bt protein expression level and its spatial distribution are widely recognized as key determinants of resistance performance in transgenic crops. Previous studies have reported significant variation in Bt protein expression among maize tissues and developmental stages. For example, Chen et al. showed that Cry1Ab/2Aj expression was highest in leaves before and after pollination [28], while Wang et al. reported a distribution pattern of “leaf > stem > root” in DBN3601T maize [29], consistent with the present study. In addition to genetic background, Bt protein expression is influenced by environmental factors (e.g., temperature and altitude) and agronomic practices (e.g., fertilization and weed management) [30,31,32]. The relatively higher Vip3Aa expression observed in this study further highlights the importance of genotype × environment interactions in regulating transgene expression. These findings suggest that the evaluation and deployment of Bt maize should consider not only genetic traits but also environmental adaptability from the perspective of plant resistance traits.

Differences in susceptibility of target pests to Bt toxins represent another key biological basis of resistance performance. In the present study, although DBN3601T and DBN9936 differed in total Bt protein expression, their resistance to *A. lepigone* did not differ significantly. This result suggests that, under the conditions of this study, toxin type may have contributed more strongly to resistance performance than total Bt protein content or pyramiding per se among the tested Bt maize events. This finding is closely related to the mode of action of Bt toxins in the insect midgut and their receptor-binding specificity, reflecting the pest-specific nature of Bt-mediated resistance traits [33,34]. Previous studies have shown that Cry toxins are highly effective against *Mythimna separata* [35], whereas Vip proteins exhibit higher activity against *Spodoptera exigua*, *S. litura*, and *Agrotis ipsilon* [36], and Cry34/35Ab1 is specifically active against *Diabrotica virgifera virgifera* [37]. Consistent with these findings, *A. lepigone* in this study showed higher sensitivity to Cry1Ab, as indicated by the lower LC_50_ value of Cry1Ab compared with Vip3Aa, which explains the superior performance of Cry1Ab-expressing maize events in simulated field cage assays. Notably, although DBN3601T expresses both Cry1Ab and Vip3Aa, its LC_50_ value was higher than that of DBN9936 expressing Cry1Ab alone. This may be because the LC_50_ values were estimated based on total Bt protein concentrations, and the inclusion of Vip3Aa, to which *A. lepigone* showed lower susceptibility, increased the total protein concentration required to reach 50% mortality. These results suggest that Cry1Ab plays a dominant role in the toxicity of DBN3601T against *A. lepigone*. Therefore, optimizing Bt toxin composition based on target pest susceptibility is essential for effective resistance deployment. However, since the cage experiments were conducted under controlled conditions, further validation under natural field conditions and pest pressure is still needed to fully evaluate the practical control efficacy of Bt maize against *A. lepigone*.

The larval developmental stage is another critical factor influencing Bt efficacy. Previous studies have shown that Bt maize is generally more toxic to early instars, whereas efficacy decreases in later instars due to physiological changes such as midgut development, receptor alteration, and enhanced detoxification capacity [38,39,40,41,42,43]. In this study, mortality decreased and survival time increased with larval instar, confirming stage-dependent differences in susceptibility. Notably, although Bt protein expression followed the pattern of leaf > stem > root, stems and roots exhibited stronger insecticidal effects against older larvae. This apparent inconsistency suggests that resistance performance is not solely determined by absolute Bt protein expression, but also by the effective exposure dose received by larvae, which depends on feeding site, feeding amount, and feeding duration. Similar feeding shifts have been reported in other noctuid pests, where early instars feed primarily on leaves, whereas later instars shift to stems, roots, or reproductive tissues [44,45]. In *A. lepigone*, early instars preferentially feed on leaves, while later instars increasingly exploit stems and roots [9], which may increase their cumulative exposure to Bt proteins in these tissues despite their lower protein concentrations. Therefore, the stronger toxicity of stems and roots to older larvae may reflect a closer match between larval feeding sites and tissue-specific Bt exposure rather than Bt protein content alone. In addition, newly hatched larvae exhibited feeding avoidance of Bt maize. Under field conditions, such avoidance behavior could potentially influence larval exposure to Bt toxins if alternative host plants or weeds are available [46,47]. This phenomenon highlights the importance of considering behavioral avoidance in resistance evaluation. Such behavioral avoidance may alter larval exposure to Bt toxins and potentially influence the selection pressure associated with resistance evolution under field conditions. However, whether adult *A. lepigone* also exhibits oviposition avoidance behavior toward Bt maize remains unclear and requires further investigation under field and agricultural production conditions. Therefore, effective deployment of Bt maize should be integrated with field management practices, such as weed control and removal of alternative hosts, to minimize behavioral escape and maximize control efficacy [48].

Despite the high efficacy observed in this study, the long-term sustainability of Bt maize is threatened by the evolution of pest resistance. Continuous selection pressure may reduce pest susceptibility to Bt toxins over time [49,50,51]. To delay resistance development, strategies such as pyramiding toxins with different modes of action and implementing refuge systems are essential [52,53]. In particular, structured refuge implementation and regular monitoring of field population susceptibility are important for reducing selection pressure and detecting early resistance evolution. Global experience from more than 25 years of Bt crop cultivation indicates that resistance management requires an integrated, systems-based approach rather than reliance on a single strategy. Continuous monitoring, strict implementation of resistance management measures, and adaptive deployment strategies are critical for ensuring the long-term effectiveness of Bt maize [54,55].

## 4. Materials and Methods

### 4.1. Insect Population and Bt Maize Materials

The insect population used in this study originated from Dongying, Shandong Province, China (118°37′13.12″ E, 37°18′8.84″ N). Adult moths of *Athetis lepigone* were captured using ground light traps and transported to the laboratory, where they were maintained with a 10% honey solution for mating and oviposition. A laboratory colony was subsequently established. Larvae from the F1–F2 generations were used for subsequent experiments. Larvae were reared on an artificial diet based on the formulation described by Jiang et al. [56], which was developed for *A. lepigone*. Newly hatched larvae were transferred to a fresh artificial diet and maintained in an insect-rearing room under controlled conditions of 25 ± 1 °C, 70 ± 5% relative humidity, and a photoperiod of 14:10 h (L:D). The diet was replaced as needed to maintain freshness, and larvae were reared until they reached the required instars for subsequent experiments.

Transgenic maize seeds were provided by Beijing DaBeiNong Biotechnology Co., Ltd., Beijing, China. Three Bt maize events expressing different Bt proteins were grown under greenhouse conditions: DBN3601T expressing Cry1Ab + Vip3Aa, DBN9936 expressing Cry1Ab, and DBN9501 expressing Vip3Aa. The conventional maize variety “Zhengdan 958” was used as the non-Bt control. All maize plants were cultivated in a greenhouse. After emergence, plants were fertilized weekly with a 0.5 g/L potassium dihydrogen phosphate solution (KH_2_PO_4_, >96.0% purity; Stanley Agricultural Group Co., Ltd., Linyi, China). Plants at the 3–4-leaf stage were used for experiments.

### 4.2. Bt Protein Expression in Larval Feeding Tissues of Bt Maize

Maize plants of uniform growth at the 3–4-leaf stage were selected from DBN3601T, DBN9936, and DBN9501 for sampling. Leaf, stem, and root tissues were collected, immediately frozen at −80 °C, and then freeze-dried for 24 h. The dried samples were ground into powder using a grinder, transferred into 50 mL centrifuge tubes, and stored at −80 °C until analysis. Bt protein contents, including Cry1Ab and Vip3Aa, in maize tissues were quantified using enzyme-linked immunosorbent assay (ELISA). Cry1Ab was measured using a QualiPlate™ kit (Envirologix, Portland, ME, USA), and Vip3Aa was measured using a YouLong ELISA kit (YouLong Biotech, Shanghai, China), according to the manufacturers’ instructions. The detection sensitivity and quantification procedures were determined according to the manufacturers’ specifications.

### 4.3. Susceptibility of Athetis lepigone Larvae to Bt Proteins

Larval susceptibility to Bt proteins was assessed using an artificial diet incorporation method. Based on Bt protein concentrations in maize leaves, freeze-dried leaf powder from DBN3601T, DBN9936, and DBN9501 was incorporated into the artificial diet at different concentrations. Bt protein contents in freeze-dried maize leaf powder were quantified by ELISA, and different amounts of freeze-dried leaf powder were incorporated into the artificial diet to obtain the target Bt protein concentrations used in the bioassays. Final concentrations were as follows: DBN3601T (Cry1Ab + Vip3Aa): 9.72, 4.86, 2.43, 1.22, and 0.61 μg∙g^−1^; DBN9936 (Cry1Ab): 5.90, 2.95, 1.47, 0.74, and 0.37 μg∙g^−1^; DBN9501 (Vip3Aa): 4.25, 2.13, 1.06, 0.53, and 0.27 μg∙g^−1^. Artificial diet containing freeze-dried leaf powder of non-Bt maize (Zhengdan 958) was used as the control. Bioassays were conducted in 24-well plates, with approximately 0.3 g diet per well and one neonate larva introduced into each well. Each concentration was replicated three times, with 24 larvae per replicate, following a completely randomized design.

All assays were conducted at 25 ± 1 °C, 70 ± 5% relative humidity, and a photoperiod of 14:10 h (L:D). Diet was replenished as needed to maintain freshness. Larval mortality was recorded after 14 days. Larvae that did not respond to gentle stimulation with a brush were considered dead. Mortality data were corrected, and LC_50_ values with 95% confidence intervals were estimated using probit analysis.

### 4.4. Bioassay of the Insecticidal Effects of Different Bt Maize Tissues on Athetis lepigone Larvae

To evaluate the insecticidal effects of different maize tissues against *Athetis lepigone* larvae, fresh leaf, stem, and root tissues were collected from Bt maize plants at the three-leaf stage and fed to first-, third-, and fifth-instar larvae. Fresh tissues were cut into approximately 1–2 cm segments and placed in 24-well plates, with one neonate larva introduced into each well. The plates were sealed with parchment paper to prevent larval escape. For third- and fifth-instar larvae, assays were conducted in 25 mL plastic cups with lids, each containing approximately 3–4 cm tissue segments, which were replaced daily with fresh tissues. The experiment included three Bt maize treatments, DBN3601T, DBN9936, and DBN9501, and a conventional maize control. For each maize treatment, three tissue types, leaves, stems, and roots, were tested in relation to first-, third-, and fifth-instar larvae. Each treatment consisted of 48 larvae per replicate, with three replicates. During the experiment, larval survival was observed and recorded daily. Larvae that showed no obvious response to gentle stimulation with a brush were considered dead.

### 4.5. Evaluation of the Feeding Preference of Athetis lepigone Larvae for Bt and Non-Bt Maize

Fresh leaves were collected from DBN3601T, DBN9936, DBN9501, and non-Bt maize plants at the 3–4-leaf stage. Fully expanded leaves at the same leaf position were selected and cut into 3 × 3 cm squares from the middle region of each leaf. In each Petri dish (12 cm diameter), one leaf piece from each maize type was randomly arranged and labeled. Ten neonate larvae starved for 4 h were released at the center of each dish. Ten replicates were conducted. The experiment was performed under dark conditions at 25 ± 1 °C and 70 ± 5% relative humidity. Larval positions were recorded at 1, 3, 5, 7, 18, and 24 h. Larvae located on a leaf were considered to have selected that maize type, whereas larvae not located on any leaf were recorded as “no choice.” The selection rate was calculated as the percentage of larvae observed on each maize leaf type relative to the total number of released larvae at each observation time point. Dishes containing dead larvae were excluded from the analysis. After 24 h, leaf images were captured, and the consumed leaf area was quantified using ImageJ software (version 1.54g; National Institutes of Health, Bethesda, MD, USA).

### 4.6. Evaluation of the Control Efficacy of Bt Maize Seedlings Against Athetis lepigone Larvae

Uniform maize plants of DBN3601T, DBN9936, DBN9501, and non-Bt maize at the 3–4-leaf stage were selected. Six plants per treatment were placed in nylon cages (50 × 50 × 50 cm, 120 mesh). Fifteen neonate larvae were introduced at the base of each plant. Experiments were conducted at 25 ± 1 °C, 70 ± 5% relative humidity, and a photoperiod of 14:10 h (L:D). Each treatment was replicated three times. Larval numbers were recorded every two days. Larvae that did not respond to gentle stimulation were considered dead.

### 4.7. Statistical Analysis

All statistical analyses were performed using SPSS 24.0, and figures were generated using GraphPad Prism 9.0. Before ANOVA, data were checked for normality and homogeneity of variance using the Shapiro–Wilk test and Levene’s test, respectively. Differences in Bt protein expression among maize events and tissues were analyzed using two-way ANOVA. Corrected mortality and larval survival time were analyzed using two-way ANOVA, with maize event and larval instar as factors. Control efficacy and feeding selection rates were analyzed using two-way ANOVA, with maize event and time as factors. Consumed leaf area was analyzed using one-way ANOVA followed by Tukey’s multiple-comparison test. Mortality curves were generated from Kaplan–Meier estimates and compared using the log-rank (Mantel–Cox) test. Larval population dynamics were fitted using a one-phase exponential decay model, and differences among fitted curves were compared using the extra sum-of-squares *F*-test. Mortality data were corrected before analysis, and dose–response relationships were analyzed using probit analysis to estimate LC_50_ values and 95% confidence intervals. Statistical significance was set at *p* < 0.05.

Corrected mortality, control efficacy, and mean survival time were calculated using Equations (1)–(3), respectively:

(1) $$\mathrm{Corrected}\;\mathrm{mortality}\;\left(\%\right)\;=\;\frac{\mathrm{mortality}\;\mathrm{in}\;\mathrm{treatment}\;-\;\mathrm{mortality}\;\mathrm{in}\;\mathrm{control}}{1\;-\;\mathrm{mortality}\;\mathrm{in}\;\mathrm{control}}\;\times \;100$$

(2) $$\mathrm{Control}\;\mathrm{efficacy}\;\left(\%\right)=\frac{\mathrm{survivors}\;\mathrm{in}\;\mathrm{control}\;-\;\mathrm{survivors}\;\mathrm{in}\;\mathrm{treatment}}{\mathrm{survivors}\;\mathrm{in}\;\mathrm{control}}\;\times \;100$$

(3) $$\mathrm{Mean}\;\mathrm{survival}\;\mathrm{time}\;(\mathrm{days})=\frac{\mathrm{total}\;\mathrm{survival}\;\mathrm{days}\;\mathrm{of}\;\mathrm{all}\;\mathrm{larvae}}{\mathrm{total}\;\mathrm{number}\;\mathrm{of}\;\mathrm{larvae}\;\mathrm{tested}}$$

## 5. Conclusions

This study systematically evaluated the resistance of Bt maize event DBN3601T, expressing Cry1Ab and Vip3Aa proteins, against *A*. *lepigone* by integrating Bt protein expression patterns with larval biological responses. DBN3601T exhibited stable Bt protein expression across different tissues and demonstrated high insecticidal activity against *A. lepigone*. Among the two toxins, Cry1Ab played a dominant role in determining resistance, as *A. lepigone* showed higher susceptibility to this protein. Although Bt protein expression varied among tissues, resistance performance was jointly influenced by larval developmental stage and feeding behavior, indicating that the efficacy of Bt maize depends on the interaction between plant resistance traits and insect ecological characteristics. These findings suggest that the development of Bt maize should prioritize optimizing toxin composition based on target pest susceptibility while ensuring stable expression across tissues to improve efficacy at different feeding sites. DBN3601T showed strong potential for controlling *A. lepigone*; however, further validation under field conditions and natural pest pressure is still needed, as field performance may be influenced by larval behavior and environmental conditions. Therefore, integrated management strategies—including ecological regulation, resistance monitoring, and resistance management—are essential to ensure the long-term effectiveness and sustainability of Bt maize.

## Figures and Tables

**Table 1:** Bt protein expression levels in leaves, stems, and roots of Bt maize events DBN3601T, DBN9936, and DBN9501 at the V4 growth stage.

Maize	Tissues	Cry1Ab (μg∙g^−1^)	Vip3Aa (μg∙g^−1^)	Total Bt Protein (μg∙g^−1^)
DBN3601T	Leaf	68.38 ± 4.32 a	53.16 ± 5.54 a	121.54 ± 5.04 Aa
	Stem	59.65 ± 2.49 ab	50.11 ± 1.03 a	109.76 ± 2.17 Ab
	Root	51.23 ± 3.30 b	15.86 ± 1.33 b	67.09 ± 2.15 Ac
DBN9936	Leaf	73.71 ± 2.61 a	/	73.71 ± 2.61 Ba
	Stem	55.11 ± 2.27 b	/	55.11 ± 2.27 Bb
	Root	46.79 ± 3.82 b	/	46.79 ± 3.82 Bb
DBN9501	Leaf	/	53.17 ± 2.91 a	53.17 ± 2.91 Ca
	Stem	/	38.02 ± 1.98 b	38.02 ± 1.98 Cb
	Root	/	10.33 ± 1.31 c	10.33 ± 1.31 Cc

Data are presented as means ± SE based on three biological replicates. V4 represents the maize vegetative growth stage with four fully expanded leaves. Different lowercase letters indicate significant differences among tissues within the same maize event, whereas different uppercase letters indicate significant differences among maize events within the same tissue (*p* < 0.05).

**Table 2:** Lethal concentrations of Bt proteins expressed in different maize events against neonate larvae of *Athetis lepigone*.

Maize	Bt Protein	LC_50_(95%FL) μg∙g^−1^	Slope ± SE	*χ* ^2^	*df*	*p*
DBN3601T	Cry1Ab + Vip3Aa	2.33(1.92–2.82) b	1.46 ± 0.15	15.95	18	0.60
DBN9936	Cry1Ab	1.05(0.86–1.25) a	1.61 ± 0.16	14.41	18	0.70
DBN9501	Vip3Aa	2.65(1.81–4.97) b	0.76 ± 0.14	10.39	18	0.92

LC_50_ represents the median lethal concentration, with 95% confidence intervals (95% FL) shown in parentheses. Slope ± SE indicates the slope of the dose–response regression and its standard error. *χ*^2^ represents the chi-square statistic for the goodness-of-fit test, *df* indicates degrees of freedom, and *p* denotes the corresponding significance level. Different lowercase letters indicate significant differences among LC_50_ values across treatments, based on non-overlapping 95% confidence intervals.

## Data Availability

Data are contained within this article.
